# Mechanisms and therapeutic opportunities in metabolic aberrations of diabetic wounds: a narrative review

**DOI:** 10.1038/s41419-025-07583-3

**Published:** 2025-04-25

**Authors:** Yuan Xiong, Samuel Knoedler, Michael Alfertshofer, Bong-Sung Kim, Dongsheng Jiang, Guohui Liu, Yuval Rinkevich, Bobin Mi

**Affiliations:** 1 Department of Orthopedics, Tongji Hospital, Tongji Medical College, Huazhong University of Science and Technology, Wuhan, 430030 China; 2 Department of Orthopedics, Union Hospital, Tongji Medical College, Huazhong University of Science and Technology, Wuhan, 430022 China; 3 Division of Plastic Surgery, Brigham and Women’s Hospital, Harvard Medical School, Boston, MA 02152 USA; 4 Institute of Regenerative Biology and Medicine, Helmholtz Zentrum München, 81377 Munich, Germany; 5 Department of Hand, Plastic and Aesthetic Surgery, Ludwig-Maximilians-University Munich, 80336 Munich, Germany; 6 Department of Plastic Surgery and Hand Surgery, University Hospital Zurich, Raemistrasse 100, 8091 Zurich, Switzerland; 7 Precision Research Centre for Refractory Diseases, Shanghai General Hospital, Shanghai Jiao Tong University School of Medicine, Shanghai, 201620 China

**Keywords:** Diabetes complications, Diseases

## Abstract

Metabolic aberrations are fundamental to the complex pathophysiology and challenges associated with diabetic wound healing. These alterations, induced by the diabetic environment, trigger a cascade of events that disrupt the normal wound-healing process. Key factors in this metabolic alternation include chronic hyperglycemia, insulin resistance, and dysregulated lipid and amino acid metabolism. In this review, we summarize the underlying mechanisms driving these metabolic changes in diabetic wounds, while emphasizing the broad implications of these disturbances. Additionally, we discuss therapeutic approaches that target these metabolic anomalies and how their integration with existing wound-healing treatments may yield synergistic effects, offering promising avenues for innovative therapies.

## Facts

- Metabolic abnormalities play a crucial role in the complex pathophysiology and challenges of diabetic wound healing.
- These disruptions, arising from the diabetic environment, initiate a cascade of events that interfere with the orderly progression of the wound repair process.
- Central to this metabolic shift are persistent hyperglycemia, insulin resistance, and imbalances in lipid and amino acid metabolism, all of which significantly hinder the body’s ability to restore normal tissue function.

## Open questions

- What impact do metabolic aberrations have on the healing of diabetic wounds?
- What are the fundamental mechanisms underlying the influence of metabolic aberrations on diabetic wound healing?
- What characterizes the features of metabolic aberrations in diabetic wounds?
- What advances have been made in metabolism-targeting therapies for diabetic wound management?

## Introduction

Diabetic wound healing presents substantial clinical challenges, marked by prolonged and intricate repair and regeneration processes. The disruption arises from the complex interaction between systemic metabolic dysfunctions and localized tissue responses. Metabolic aberrations inherent to diabetes critically affect the delicate and precise mechanisms of wound healing, ultimately leading to impaired tissue repair [1]. The metabolic state plays a crucial role in wound healing, acting as the driving force behind the intricate coordination of tissue repair processes [2]. It provides energy, substrates, and signaling molecules essential for the progression of healing events. However, in diabetes, this delicate equilibrium is disrupted, giving rise to metabolic anomalies that significantly impede the healing process.

Wound healing is a complex, highly coordinated process that involves several overlapping stages: hemostasis, inflammation, proliferation, and remodeling [3, 4]. Initially, when tissue is injured, the body responds by activating the clotting cascade to halt bleeding, which forms a temporary matrix for cell migration. This is followed by the inflammatory phase, where immune cells such as neutrophils and macrophages infiltrate the wound site to clear debris and prevent infection. As inflammation subsides, the proliferative phase begins, characterized by the formation of new blood vessels (angiogenesis), the proliferation of fibroblasts, and the deposition of extracellular matrix components like collagen. During this stage, epithelial cells also migrate to cover the wound surface, facilitating tissue repair. The final phase, remodeling, involves the maturation and realignment of collagen fibers, leading to increased tissue strength and elasticity over time. This dynamic process can be disrupted in chronic conditions like diabetes, where impaired immune responses, poor circulation, and metabolic imbalances hinder efficient healing, resulting in persistent, non-healing wounds [5]. Diabetes is characterized by hyperglycemia, insulin resistance, and imbalances in lipid and amino acid metabolism [6, 7]. Collectively, these factors compromise the intricate mechanisms that regulate wound healing. Hyperglycemia, considered a hallmark of diabetes, results in a series of biochemical alterations that both directly and indirectly impede the wound-healing process [8]. Elevated glucose levels impair cellular functions, weaken immune responses, disrupt fibroblast activity and collagen synthesis, and intensify oxidative stress within the wound microenvironment.

Alongside hyperglycemia, insulin resistance-a hallmark of type 2 diabetes-further exacerbates the spectrum of metabolic disruptions, complicating the wound healing process [2]. Typically, insulin signaling is central to cell proliferation, migration, and tissue regeneration during wound healing [9]. However, in individuals with insulin resistance, this signaling pathway is compromised, resulting in impaired cellular responses. In addition, increased levels of inflammatory molecules, characteristic of insulin resistance, foster a pro-inflammatory environment at the wound site, thereby disrupting the delicate equilibrium necessary for effective wound healing [10, 11]. Notably, previous studies indicate that insulin resistance can disrupt the signaling of essential growth factors, including the insulin-like growth factor (IGF), pivotal for tissue regeneration [12]. The interplay between impaired insulin signaling, chronic inflammation, and altered growth factor responses collectively impedes the normal progression of wound healing in insulin-resistant individuals, often leading to delayed and compromised tissue repair.

Moreover, during diabetic wound healing, alterations in lipid and amino acid metabolism also contribute to delayed healing and poor tissue repair [7, 13]. Elevated lipid peroxidation, driven by increased oxidative stress, generates toxic lipid peroxides, triggering a form of cell death known as ferroptosis [14]. This iron-dependent process exacerbates oxidative damage and inflammation, delaying the wound-healing process. Accordingly, amino acid metabolic changes, particularly arginine deficiency, compromise nitric oxide (NO) synthesis, a vital mediator of wound repair due to its central role in vasodilation, angiogenesis, and immune cell function [13]. Additionally, glutamine metabolism in activated macrophages can overwhelm mitochondria, leading to toxic metabolite accumulation and inflammation [15].

In this review, we provide an overview of the complex mechanisms underlying metabolic alterations in diabetic wounds, with the goal of elucidating the broad and multifaceted consequences of these metabolic anomalies. Additionally, we examine therapeutic approaches aimed at targeting these metabolic changes, emphasizing the potential for synergistic effects when integrated with existing wound-healing treatments.

## Metabolic aberrations in diabetic wound

**Fig. 1:**
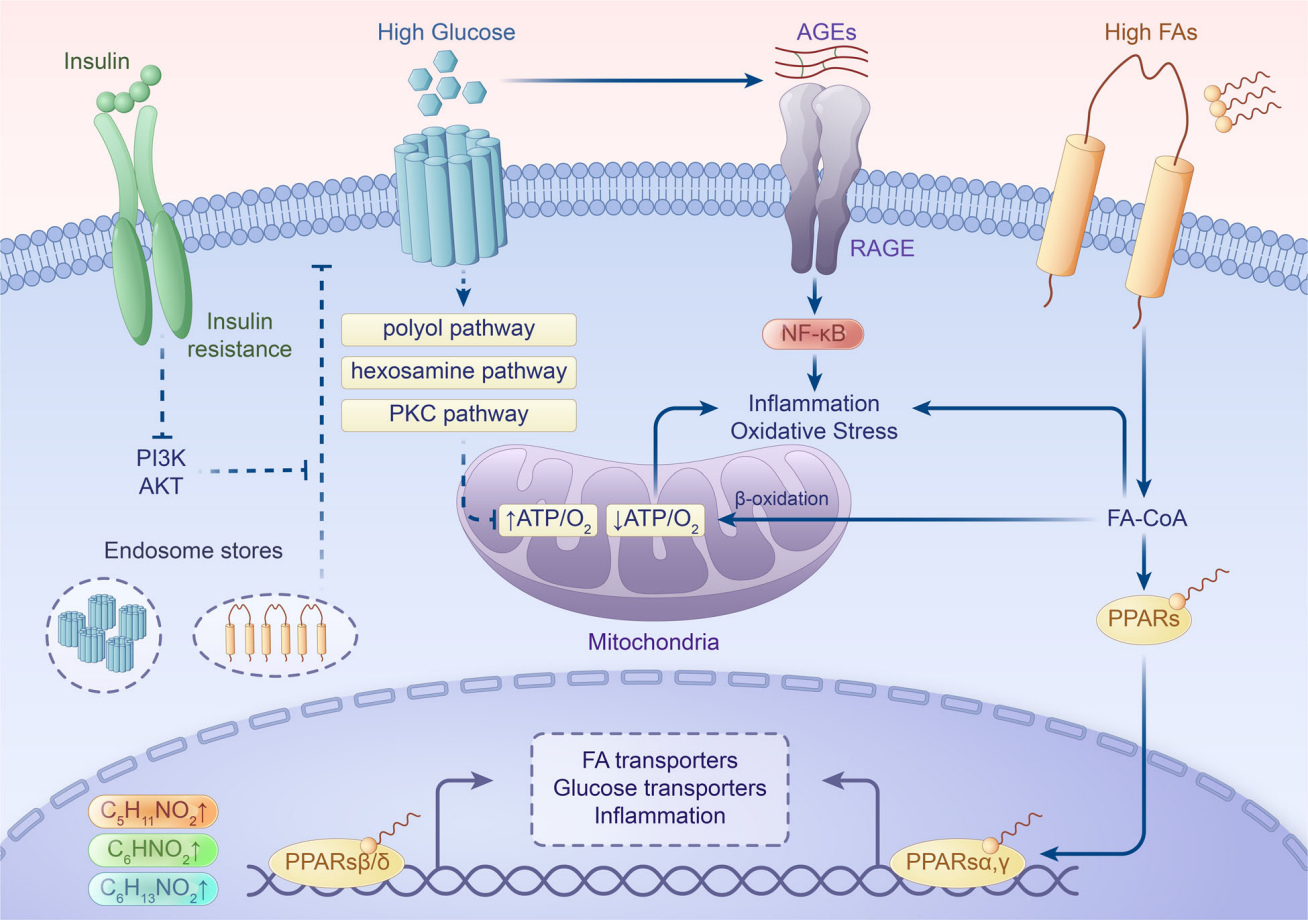
Key metabolic signaling pathways in diabetic wounds. Hyperglycemia exacerbates inflammation and oxidative stress, and disrupts energy production by promoting a metabolic shift towards glucose use. Alongside hyperglycemia, insulin resistance compounds the complexity of metabolic disturbances. Insulin resistance diminishes cells’ capacity to efficiently utilize glucose, further perpetuating the metabolic imbalances that impede the healing process. In addition, the dysregulation of lipid and amino acid metabolism additionally deepens the metabolic challenges of diabetes in wound healing.

Metabolic aberrations in diabetic wounds include a range of disturbances in cellular and molecular processes, impeding effective wound healing [16] (Fig. 1). In acute wound healing, regulated metabolic processes ensure the timely activation and migration of immune cells, fibroblasts, and endothelial cells to facilitate inflammation, angiogenesis, and tissue remodeling [17]. However, in chronic wounds, such as those observed in diabetic patients, metabolic dysregulation impairs these processes at multiple stages [18]. Hyperglycemia leads to the formation of advanced glycation end-products (AGEs) that alter extracellular matrix (ECM) structure, trapping immune cells and disrupting their ability to clear debris and pathogens efficiently. This contributes to prolonged and excessive inflammation, as macrophages remain in a pro-inflammatory state, failing to transition into a reparative mode. Insulin resistance exacerbates this by reducing glucose uptake in cells, leading to impaired energy production needed for cell migration and proliferation [1, 19]. This limits the activity of fibroblasts and endothelial cells, slowing the deposition of extracellular matrix and the formation of new blood vessels, critical for oxygen and nutrient delivery during wound repair.

Moreover, dysregulated lipid metabolism results in the accumulation of toxic lipid intermediates, which further impair immune cell function and promote a chronic inflammatory environment [20]. The altered amino acid metabolism, particularly reduced arginine bioavailability, impairs nitric oxide production, an essential mediator for vasodilation and angiogenesis. As a result, angiogenesis is delayed, compromising blood vessel formation and leading to hypoxic conditions that hinder tissue repair. Tissue remodeling is also affected, as fibroblasts exhibit reduced collagen synthesis and improper extracellular matrix remodeling due to both hyperglycemia and impaired insulin signaling [21]. Consequently, wounds become stuck in a persistent inflammatory phase, with inadequate tissue regeneration and prolonged remodeling.

### Hyperglycemia

Hyperglycemia, a hallmark of diabetes and metabolic disorders, arises from a complex interplay of molecular interactions prompted by metabolic shifts. This cascade of events is driven by persistently elevated hyperglycemia, which activates a network of interconnected pathways that contribute to sustained high blood glucose levels. A critical consequence of chronic hyperglycemia is the excessive production of reactive oxygen species (ROS) within cells [22]. ROS, including superoxide anions and hydrogen peroxide, are highly reactive molecules capable of causing cellular damage. Excessive ROS production, driven by hyperglycemia, overwhelming the cell’s antioxidant defenses, leading to oxidative stress [23].

Oxidative stress triggers a cascade of molecular reactions that exacerbate hyperglycemia and its associated complications [24]. A prominent pathway triggered by oxidative stress is the polyol pathway [23]. In this pathway, glucose is metabolized to sorbitol by the enzyme aldose reductase, consuming excess NADPH in the process. This depletion of NADPH impairs the cell’s ability to regenerate reduced glutathione, a critical antioxidant, thereby leading to an exacerbation of oxidative stress. Another metabolic pathway affected by chronic hyperglycemia is the hexosamine pathway. Here, glucose is diverted toward the production of molecules like uridine diphosphate N-acetylglucosamine (UDP-GlcNAc) [25, 26]. This alteration disrupts the normal post-translational modification of proteins by *O*-GlcNAcylation, impacting various cellular functions, including insulin signaling.

Additionally, hyperglycemia promotes overactivation of protein kinase C (PKC) isoforms [23]. PKC is a family of signaling enzymes involved in numerous cellular processes. When overactive, PKC isoforms contribute to insulin resistance, reducing the cells’ efficiency in responding to insulin and taking up glucose, further exacerbating hyperglycemia. AGEs are another consequence of prolonged hyperglycemia [23]. These ROS are generated when glucose molecules irreversibly bind to proteins, altering their structure and function. When AGEs interact with cell surface receptors, particularly the receptor for advanced glycation end-products (RAGE), they trigger inflammatory responses that contribute to tissue damage.

Moreover, epigenetic modifications, driven by hyperglycemia, play a critical role in the development of metabolic alterations [27]. Epigenetic modifications, such as DNA methylation and histone modifications, can affect gene expression patterns. In the context of hyperglycemia, these modifications often lead to the suppression of genes involved in the antioxidant defense systems, further exacerbating oxidative stress and perpetuating the cycle of metabolic dysfunction [22, 23].

Excessive ROS accumulation in cells causes mitochondrial damage, a central event in the progression of metabolic complications [28]. Mitochondria are the cellular powerhouses responsible for the production of adenosine triphosphate (ATP), the cell’s primary energy source [29]. When mitochondria are damaged, ATP production is compromised, contributing to cellular dysfunction and metabolic disturbances. Mitochondrial dysfunction, in turn, leads to cellular apoptosis, or programmed cell death [30]. Mitochondrial energy metabolism plays a critical role in cellular processes, and its dysfunction is a hallmark of impaired wound healing, particularly in diabetic wounds [31]. Two central metabolic pathways, oxidative phosphorylation (OXPHOS) and the tricarboxylic acid (TCA) cycle, are essential for generating ATP, the primary energy currency of the cell [32]. In healthy cells, the TCA cycle occurs within the mitochondrial matrix, where acetyl-CoA, derived from carbohydrates, fats, and proteins, enters a series of enzymatic reactions that produce reduced coenzymes NADH and FADH2. These coenzymes then donate electrons to the electron transport chain (ETC) during OXPHOS, generating a proton gradient across the inner mitochondrial membrane, ultimately driving ATP production.

In diabetic wounds, however, mitochondrial function is often compromised due to hyperglycemia-induced metabolic stress, leading to impaired TCA cycle activity and reduced OXPHOS efficiency [33]. This metabolic abnormality decreases ATP production, depriving cells of the energy required for essential repair processes such as cell proliferation, migration, and collagen synthesis. The disrupted energy homeostasis severely hampers the wound healing cascade, particularly in the early stages of inflammation and granulation tissue formation [34].

Moreover, impaired mitochondrial metabolism in diabetic wounds is closely linked to increased oxidative stress. As OXPHOS becomes less efficient, electron leakage from the ETC leads to the excessive generation of ROS. This oxidative burden not only prolongs the inflammatory phase of wound healing but also promotes cellular senescence and apoptosis, further delaying tissue repair [35, 36]. The metabolic inflexibility seen in diabetic wounds, where cells struggle to switch between fuel sources due to mitochondrial dysfunction, exacerbates this situation. Additionally, mitochondrial fragmentation and reduced biogenesis are often observed, which further deteriorate the cell’s ability to regenerate functional mitochondria, thus prolonging energy deficits [34].

### Insulin resistance

Insulin resistance is acquired through a complex interplay of chronic inflammation, impaired insulin signaling, and altered lipid metabolism, all of which are interconnected [37]. Chronic inflammation, often triggered by obesity or metabolic stress, plays a central role in the development of insulin resistance [38]. Adipose tissue, when overburdened, releases pro-inflammatory cytokines such as tumor necrosis factor-alpha (TNF-α) and interleukin-6 (IL-6), which activate inflammatory pathways like nuclear factor-kappa B (NF-κB) [39]. These pathways disrupt normal insulin signaling by impairing the insulin receptor substrate (IRS) proteins, crucial for transmitting insulin’s signal inside cells [40]. The inflammation-induced interference with IRS proteins blocks downstream activation of the phosphatidylinositol 3-kinase (PI3K) and AKT pathway, which is essential for glucose uptake in muscle and fat cells, thus leading to reduced cellular sensitivity to insulin [41].

Altered lipid metabolism further contributes to insulin resistance. In conditions of excess fat, free fatty acids (FFAs) accumulate in the bloodstream and are taken up by non-adipose tissues like muscle and liver [42]. The buildup of FFAs in these tissues leads to the formation of toxic lipid intermediates, such as diacylglycerol (DAG) and ceramides. These lipid byproducts activate stress-related kinases, such as PKC, which also inhibit insulin signaling by phosphorylating IRS proteins at sites that reduce their effectiveness [43]. This combination of chronic inflammation, impaired insulin signaling, and lipid toxicity results in decreased glucose uptake by cells, forcing the pancreas to produce more insulin.

This dyslipidemic state is characterized by elevated low-density lipoprotein (LDL) and decreased high-density lipoprotein (HDL), further exacerbating inflammation [44]. The inflammatory cytokines produced in response to lipid accumulation, compromise endothelial cell function, impairing their ability to respond to pro-angiogenic signals. This diminished angiogenic response is particularly detrimental, as it reduces the formation of new blood vessels essential for delivering oxygen and nutrients to healing tissues, which is critical in wound repair.

In the context of aging, the effects of insulin resistance and altered lipid metabolism are magnified. Aging is associated with a natural decline in insulin sensitivity, which, when combined with pre-existing insulin resistance, accelerates the onset of dyslipidemia and chronic inflammation [45]. This interplay leads to a compounded effect on wound healing, as older adults already experience slower repair processes [46]. Additionally, chronic inflammation, often termed “inflammaging”, exacerbates the situation by perpetuating a cycle of immune dysfunction and tissue degeneration, further impairing angiogenesis and tissue remodeling [45, 47].

The relationship between these interconnected processes illustrates the complexity of wound healing complications in diabetic patients. Chronic inflammation and dysregulated lipid metabolism not only hinder angiogenesis and the normal remodeling of the extracellular matrix but also create an environment that favors persistent inflammation and tissue breakdown. Addressing these multifaceted interactions is crucial for developing comprehensive therapeutic strategies that can effectively enhance wound healing outcomes in diabetic individuals, particularly as they navigate the challenges posed by aging and chronic inflammatory states.

### Dysregulation of lipid and amino acid metabolism

Diabetes induces substantial disturbances in lipid and amino acid metabolism, arising from complex molecular mechanisms that collectively result in metabolic imbalance. In the field of lipid metabolism, diabetes frequently gives rise to dyslipidemia, characterized by elevated levels of triglycerides and LDL cholesterol, coupled with decreased levels of protective HDL cholesterol [48]. Key enzymes and transcription factors governing lipid synthesis, transport, and storage such as peroxisome proliferator-activated receptors (PPARs) and sterol regulatory element-binding proteins (SREBPs) assume critical roles in orchestrating these changes, promoting increased hepatic production of triglycerides and hindering LDL clearance [49, 50].

Diabetes further disrupts lipid metabolism by promoting lipolysis in adipose tissue due to insulin resistance [51]. This heightened lipolytic activity releases great amounts of fatty acids into the bloodstream. While these fatty acids can serve as an energy source under normal conditions, the excess contributes to insulin resistance in muscle and liver tissue in diabetic patients, thereby further disrupting glucose metabolism [52]. Additionally, these excess fatty acids may accumulate within non-adipose tissues, culminating in lipotoxicity and inflammation, further impeding overall metabolic health.

Amino acid metabolism, especially of branched-chain amino acids (BCAAs) such as leucine, isoleucine, and valine, is also affected in diabetes. Individuals with insulin resistance and type 2 diabetes commonly exhibit elevated levels of BCAAs [52]. The exact molecular mechanisms responsible for this elevation remain unclear; however, they are believed to involve changes in amino acid transporters and enzymes that regulate amino acid metabolism [53]. Elevated BCAAs have been causally linked to insulin resistance and are intricately associated with impaired insulin signaling pathways.

Moreover, diabetes is characterized by chronic low-grade inflammation and oxidative stress, both of which have detrimental implications for metabolic pathways, including lipid and amino acid metabolism [54]. Inflammatory cytokines, such as TNF-α and IL-6, are elevated in diabetes and exert inhibitory effects on insulin signaling pathways, thereby perpetuating insulin resistance [55, 56]. Concurrently, these cytokines promote lipolysis, fostering the release of more fatty acids into circulation [53, 57]. Additionally, the pro-inflammatory milieu can impact amino acid metabolism, further destabilizing metabolic homeostasis.

Mitochondria, the cellular organelles responsible for energy production, are pivotal in lipid and amino acid metabolism. In diabetes, mitochondrial dysfunction often occurs, leading to decreased energy production, increased oxidative stress, and compromised metabolic processes [51, 58]. This dysfunction extends to the breakdown of fatty acids and amino acids for energy production, further exacerbating metabolic derangement [58].

Finally, genetic and epigenetic factors play a critical role in regulating the complex molecular mechanisms underlying lipid and amino acid metabolism in diabetes. Specific genetic variations in genes related to lipid metabolism and insulin signaling pathways can heighten the risk of dyslipidemia and insulin resistance, thereby exacerbating metabolic disturbances [8]. Moreover, epigenetic modifications, encompassing DNA methylation and histone acetylation, can modulate gene expression patterns in response to environmental factors, including dietary choices and physical activity levels, further shaping the metabolic landscape in diabetes [59].

## Effect of metabolic aberrations on wound healing

**Fig. 2:**
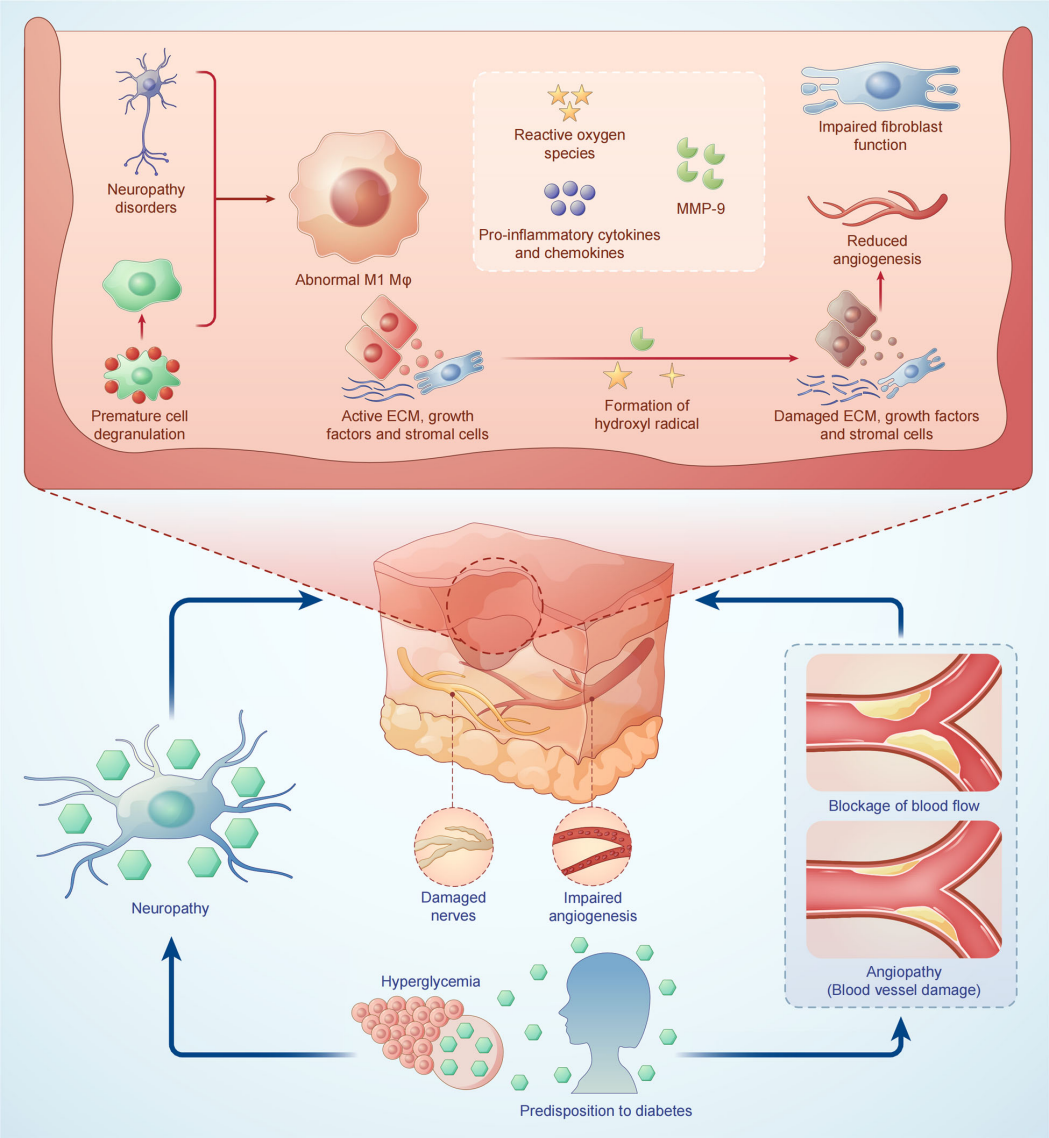
Schematic representation of metabolic aberrations in wound healing impairment. Prolonged hyperglycemia promotes oxidative stress, resulting in cellular damage and impaired tissue regeneration. Simultaneously, it induces chronic inflammation, hampering the immune response and prolonging the transition to the proliferative phase of wound healing. Dysregulated angiogenesis results in disrupted nutrient delivery, thereby further impairing reparative processes. Diabetic neuropathy delays injury detection and compromises muscle function, hindering wound closure. A combination of disrupted cell signaling, continuous inflammation, and oxidative damage collectively impair reepithelialization.

Metabolic aberrations in diabetes significantly disrupt wound healing. Chronic inflammation impedes immune response and prolongs the transition to the proliferative phase of wound healing. Elevated glucose levels induce oxidative stress, thereby damaging cells and impairing tissue regeneration. The process of angiogenesis, essential for nutrient delivery through the formation of new blood vessels, is dramatically impaired due to a disrupted glucose metabolism. Compounding this, diabetic neuropathy both delays the sensoric detection of injuries and weakens muscle function essential for wound closure. Altered cell signaling, inflammation, and oxidative stress hinder reepithelialization. Addressing these metabolic factors through comprehensive strategies, including glycemic control and inflammation management, is crucial for improving diabetic wound healing outcomes and reducing complications (Fig. 2).

### Immune microenvironment alteration

Diabetic wounds present unique challenges due to alterations in the immune microenvironment, resulting from a complex interplay of factors that significantly impact the wound-healing process. A key metabolic change in diabetic wounds is the decreased glycolytic activity observed in immune cells [60, 61]. Reduced glycolysis impairs the functional capacity of these immune cells, leading to reduced migration capabilities and diminished production of cytokines essential for effective wound healing.

For instance, macrophage metabolism plays a significant role in shaping their function during wound healing, influencing their ability to switch between pro-inflammatory and anti-inflammatory phenotypes [62]. During the initial inflammatory phase, M1 macrophages predominantly rely on glycolysis for rapid ATP production, enabling them to generate inflammatory cytokines and ROS necessary for pathogen clearance and debris removal. This glycolytic shift supports their energy-intensive tasks, but prolonged M1 activation, particularly in diabetic wounds, can lead to excessive inflammation, impairing healing [63]. As wound healing progresses, macrophages are required to transition to the M2 phenotype, which relies more on OXPHOS and FAO for energy. These metabolic pathways allow M2 macrophages to perform functions essential for tissue repair, such as collagen deposition, angiogenesis, and ECM remodeling [64]. This shift from glycolysis to OXPHOS is crucial for reducing inflammation and promoting wound resolution. However, in diabetic wounds, metabolic dysfunction often traps macrophages in a sustained M1 state, preventing the shift to OXPHOS and delaying the healing process [63]. The hyperglycemic and oxidative stress conditions in diabetes exacerbate this metabolic imbalance, prolonging inflammation and impairing tissue regeneration.

Hyperglycemia, a hallmark feature of diabetes, directly contributes to the inhibition of the PI3K/AKT signaling pathway by further elevating ROS levels. This interference with the signaling cascade in diabetic corneal epithelial cells not only weakens their overall functionality but also exacerbates a condition known as diabetic keratopathy, adding to wound healing challenges [65]. Moreover, the accumulation of effector T cells, a primary source of pro-inflammatory cytokines like TNF-α perpetuates chronic inflammation and delays repair [61].

A central aspect of wound healing is efferocytosis, the process by which dying cells are removed from the wound site [60]. In diabetes, the extended presence of dying cells amplify inflammation and hinder tissue repair. Notably, enhancing efferocytosis by targeting the SLC7A11 membrane transporter in dendritic cells can promote efficient removal of dying cells and thus foster a more supportive environment for wound healing [60].

Furthermore, the regulation of cell metabolism in diabetic wounds is linked to growth factors such as EGFR ligands, IGF1, and neurokinin 1 (NK-1) [65]. These growth factors have the capacity to enhance cell proliferation, migration, and anti-apoptotic mechanisms within corneal epithelial cells. However, hyperglycemia in diabetes reduces their efficacy, further hindering the wound-healing process.

In sum, these metabolic changes culminate in chronic inflammation, diminished immune cell function, and prolonged wound healing in diabetic individuals. Understanding and addressing these metabolic intricacies offer promising avenues for therapeutic interventions aimed at improving the wound-healing process in diabetic patients, potentially mitigating the burdensome complications associated with diabetic wounds.

### Oxidative damage

Oxidative damage is a hallmark of the protracted wound-healing process observed in diabetic ulcers. In individuals with diabetes, elevated blood glucose levels continuously influence cells, setting the stage for a series of cascading events. The primary consequence of hyperglycemia is the abnormal metabolism of glucose within these cells [66]. As glucose undergoes metabolic pathways such as glycolysis and the pentose phosphate pathway (PPP), it inevitably leads to an increased production of ROS, including superoxide radicals and hydrogen peroxide [67, 68]. While ROS are essential for cellular signaling, excessive generation jeopardizes cellular integrity and function. Elevated glucose metabolism places a substantial strain on the mitochondria. Consequently, the mitochondria’s electron transport chain, responsible for energy production, becomes compromised, further inducing ROS production and compounding oxidative stress [35].

The cell’s natural antioxidant defense mechanisms, designed to counteract ROS, are overwhelmed, allowing these highly reactive molecules to damage cellular components [68]. Oxidative stress damages proteins, lipids, and DNA, leading to functional impairments that exacerbate disruptions in cellular metabolism. A notably adverse outcome of oxidative damage is the occurrence of lipid peroxidation, where ROS instigate the oxidation of lipids within cell membranes, setting off a chain reaction of deleterious consequences. Lipid peroxidation, arising from oxidative stress, induces the breakdown of cell membrane lipids, compromising the structural resilience of cells and, consequently, impeding essential cellular processes. This process additionally gives rise to noxious byproducts, notably reactive aldehydes, which exacerbate cellular injury and incite inflammatory responses [67].

Moreover, the persistent hyperglycemic milieu in diabetes disrupts iron metabolism, resulting in excess free iron in the bloodstream. This iron overload acts as a catalyst for a recently discovered form of cell death known as ferroptosis, characterized by iron-dependent lipid peroxidation [67]. Ferroptosis adds an additional layer of complexity to the cellular response to oxidative stress in diabetic wounds. An intriguing facet of metabolic abnormalities in diabetic fibroblasts lies in the regulation of ferroptosis, although its precise consequences remain a subject of ongoing debate. Notably, impaired ferritinophagy has been observed in senescent fibroblasts within diabetic wounds, rendering them resistant to ferroptosis. This resistance to ferroptosis is closely linked to the reduced expression of NCOA4, a key factor that influences ferritinophagy, which is consistently observed in both diabetic wound tissue and high glucose-induced senescent fibroblasts [69]. Conversely, *i*n vitro studies reveal that fibroblasts exposed to high glucose concentrations exhibit elevated levels of ferroptosis-associated proteins and display reduced survival and migration. These deleterious effects of high glucose are significantly mitigated by treatment with the ferroptosis inhibitor, ferrostatin-1 (Fer-1) [14].

Additionally, oxidative stress and lipid peroxidation byproducts can activate inflammatory pathways, exacerbating tissue damage further. The resulting inflammation creates an inhospitable microenvironment at the wound site, impeding the natural wound-healing process. Neovascularization, essential for providing nutrients and oxygen to injured tissue, becomes compromised. Consequently, the formation of new blood vessels is hindered, and tissue repair is significantly delayed [70]. These detrimental processes culminate in chronic wounds that resist healing, a hallmark of diabetic ulcers.

### Altered cellular state and impaired functions of fibroblasts

Fibroblasts constitute a vital component of the skin and play pivotal roles in the intricate process of wound repair [71, 72]. Among the various stromal cell types involved, fibroblasts emerge as the linchpin of wound healing. Notably, the dysregulation of fibroblast activity stands out as a defining pathological feature in diabetic wounds. The orchestrated functions of fibroblasts and their gradual differentiation into myofibroblasts hold the key to wound closure and the remodeling of the ECM. It is worth noting that distinct populations of fibroblasts inhabit different layers of the skin: papillary dermal fibroblasts are responsible for regeneration, while reticular fibroblasts tend to contribute to scar formation [73–75]. Recent research has revealed a significant role for fibroblasts dwelling in the deepest skin layer, the subcutaneous fascia, as the primary drivers of deep wound repair and scar formation [76, 77]. These subcutaneous fascia fibroblasts employ a network of intercellular adhesion and communication mechanisms, such as N-cadherin-based adherens junctions, Connexin-43-based gap junctions, and p120-catenin, facilitating their directed collective migration to the wound site [78–80]. The intriguing aspect of their involvement in wound healing lies in their transition through sequential cellular states, culminating in the execution of significant functions. These stages involve progression from CD201 (Procr)-expressing progenitor cells to podoplanin (PDPN)-expressing pro-inflammatory fibroblasts, further transitioning into phospho-STAT3-expressing proto-myofibroblasts, and ultimately reaching the myofibroblast state marked by RUNX2 expression [81].

Cellular metabolism stands as the main pillar underlying all biological processes, including the intricate choreography of wound repair. Recent findings emphasize the critical role of metabolic reprogramming within fibroblasts at the wound site, a phenomenon vital for fibroblast activation and state transitions, ensuring a harmonious healing process [82, 83]. Notably, the metabolites themselves serve as potent signaling molecules within the milieu of wound fibroblasts. For instance, intermediates and derivatives of the TCA cycle, including succinate, itaconate, citrate, αKG, 2-hydroxyglutarate isomers, fumarate, and acetyl-CoA, play instrumental roles to instruct fibroblasts during repair processes [84]. In the context of diabetes, fibroblasts often exhibit a metabolic shift towards increased glycolysis. This shift is associated with the accumulation of abnormal metabolites, including elevated l-lactate, contribute to cytotoxicity and a detrimental functional impairment in diabetic fibroblasts [85].

The altered metabolic state in diabetic fibroblasts manifests as a hindrance to their ability to efficiently migrate and proliferate, two pivotal processes in wound repair. High-glucose treatment or glyoxal induced glycation, which mimics chronic hyperglycemia, have been shown to suppress JNK activity inhibit bFGF signaling [86]. The result of this inhibition is a significant reduction in the proliferation and migration capacity of cultured human dermal fibroblasts [87]. Moreover, the altered sphingolipids metabolism led to the accumulation of an atypical class of 1-deoxy-sphingolipids, which in turn inhibits the migration of fibroblasts, as evidenced in studies involving 3T3 fibroblasts [88]. Pyruvate dehydrogenase kinase 4 (PDK4), a pivotal enzyme responsible for regulating glucose metabolism, emerges as a key player in fibroblast function during wound repair. Sufficient PDK4 expression in fibroblasts has been shown to be essential for a normal wound-healing process by promoting fibroblast proliferation and migration. In contrast, diabetic fibroblasts exhibit significantly lower expression of PDK4. Interestingly, supplementing PDK4 has been demonstrated to accelerate wound healing in diabetic mice [89], underscoring the therapeutic potential of targeting metabolic pathways to mitigate the impaired wound repair process in diabetes.

The altered metabolism in diabetic fibroblasts extends its influence to the intricate processes of fibroblast cell state transition and differentiation during wound healing, as well as their clearance post-repair. In diabetes, insulin resistance poses a significant hurdle as it prevents the down-regulation of 11-beta-hydroxysteroid dehydrogenase 1 (11β-HSD1) in fibroblasts. This lack of regulation disrupts control over glucocorticoid availability, culminating in elevated endogenous glucocorticoid levels within diabetic skin. These elevated levels have been strongly associated with impaired wound healing in patients with diabetes [90]. Moreover, fibroblasts from diabetic patients and diabetic mouse models display reduced myofibroblast maturation [91]. This diminished maturation process has been linked to a significant increase in the expression of PKCδ [92].

Notably, the metabolic alterations in wound fibroblasts during diabetic wound healing are not solely dictated by nutrient availability; they are also intricately controlled by the chronic inflamed immune microenvironment. These factors are linked to a fragmented TCA cycle, a shift towards aerobic glycolysis, and the activation of inflammatory pathways [82]. A crucial regulator in this metabolic interplay is the transcription factor hypoxia-inducible factor 1a (HIF-1α), known to orchestrate the transcriptional programs of glycolytic metabolism and pro-inflammatory cytokine production [93]. Succinate, which accumulates as a consequence of a disrupted TCA cycle under inflammatory conditions, significantly impacts HIF-1α stability [94]. Recent research by our group and others has illuminated that gene expression and protein stability of HIF-1α serve as key factors driving the fibroblast state transition from the pro-inflammatory state marked by podoplanin (PDPN) expression to the myofibroblast state [81, 89].

The metabolic alterations in fibroblasts extend their influence on the process of ECM remodeling. Elevated glucose levels have been linked to lactic dehydrogenase (LDH) leakage, culminating in reduced levels of exendin-4. Exendin-4 is a glucagon-like peptide-1 analog, is responsible for inducing glycosaminoglycan (GAG) synthesis in fibroblasts under normal conditions. Consequently, GAG production in diabetic fibroblasts is significantly hindered [95]. Furthermore, the high glucose-induced escalation of IL-7 release from dermal fibroblasts has been associated with the inhibition of angiogenesis, a process crucial for effective wound healing [96].

Despite the valuable insights discussed above, it is remarkable that research into the impact of metabolic alterations on fibroblast functions during wound healing under diabetic conditions is still in its early stages. The majority of studies have primarily relied on cultured fibroblasts, often derived from a limited number of diabetic patient donors or exposed to artificial hyperglycemic in vitro conditions, often with over-simplified scratch assays used to model the complex cascade of wound healing events. To deepen our understanding, future research endeavors should strive to catch up by employing dedicated in vivo models combined with cutting-edge techniques such as single-cell RNA sequencing and other high-definition approaches. These methods will enable researchers to elucidate the intricate molecular mechanisms underlying metabolic regulations in each distinct fibroblast subset and cellular state throughout the wound healing process, in both healthy and diabetic conditions.

### Impaired angiogenesis

Hyperglycemia significantly disrupts endothelial cell function, which is critical for the formation of new blood vessels during wound healing [97]. Elevated glucose levels lead to the accumulation of AGEs in endothelial cells, causing structural and functional alterations in the vasculature. This oxidative damage impairs NO production, a key signaling molecule involved in vasodilation and angiogenesis. Reduced NO availability hinders endothelial cell migration, proliferation, and their ability to form tubular structures, which are essential for new blood vessel formation [98]. Furthermore, hyperglycemia disrupts angiogenic signaling pathways, including VEGF signaling, which plays a significant role in promoting endothelial cell survival and angiogenesis [99].

The activation of pro-inflammatory pathways, such as NF-κB and the overexpression of inflammatory cytokines like TNF-α, further impair endothelial function by promoting cellular damage and apoptosis [100]. The resulting endothelial dysfunction is characterized by increased vascular permeability, poor responsiveness to angiogenic cues, and an overall reduction in neovascularization. Additionally, hyperglycemia-induced inflammation promotes the formation of dysfunctional, leaky blood vessels, further compromising tissue oxygenation and nutrient delivery to the wound site [101].

The ECM, a structural scaffold necessary for efficient angiogenesis, undergoes abnormal remodeling in diabetes, creating barriers that impede endothelial cell migration and blood vessel formation [102]. Elevated levels of specific microRNAs (miRNA) in diabetic tissues further suppress genes that promote angiogenesis and disturb essential cellular metabolic activities for angiogenesis [103]. Additionally, hypoxia exacerbates these challenges. By restricting oxygen delivery to the wound site, hypoxia impairs cellular metabolism and angiogenesis.

Consequently, impaired angiogenesis in hyperglycemic conditions contributes to delayed wound healing, as the wound environment becomes hypoxic and nutrient-deprived, which is essential for proper tissue repair. In diabetic wounds, the combination of endothelial dysfunction, reduced angiogenic signaling, and chronic inflammation leads to persistent vascular deficits, preventing effective revascularization and significantly contributing to chronic, non-healing wounds.

### Neuropathy

In the cornea, there exists a dynamic crosstalk between sensory nerve endings and dendritic cells. This interaction is crucial for maintaining epithelial homeostasis, as these cells synergistically secrete growth factors, neurotrophic factors, and cytokines, which support and regulate the function and survival of adjacent cells [104]. In turn, sensory nerves release neuropeptides that counter inflammation and promote epithelial wound healing, while immune cells secrete crucial neurotrophic and growth factors to support nerve and epithelial cells [104]. However, diabetes critically perturbs these symbiotic relationships, manifesting as reduced epithelial proliferation, sensory neuropathy, and a diminished density of dendritic cells. Clinically, these disruptions manifest as markedly delayed wound healing and regeneration of sensory nerves following injury.

Diabetic neuropathy further impacts cellular metabolism by influencing critical pathways such as the mechanistic target of rapamycin (mTOR) and AMP-activated protein kinase (AMPK) [105]. The activation of AMPK is essential for the regulation of various cellular processes, including autophagy and glucose homeostasis, and is susceptible to modulation by factors within the Wnt signaling pathway.

Of note, cell-based treatments are promising in addressing the fundamental causes of diabetic neuropathy within wound sites [104]. Additionally, researchers explored the modulation of growth factors and cytokines, aiming to restore the delicate balance disrupted by diabetes [106]. Novel strategies targeting negative regulators of Schwann cell differentiation further offer potential avenues to enhance the regenerative capacity of peripheral nerves [107, 108]. Antioxidant interventions, while facing challenges, remain a potential route to mitigate oxidative stress-induced cellular damage [109, 110]. By exploring these cellular and metabolic mechanisms, researchers aim to discover optimal therapies and interventions to reduce the impact of diabetic neuropathy on wound healing.

### Compromised epithelial regeneration

The regeneration of epithelial tissues, as highlighted in various studies, can be examined from a cellular metabolism perspective, revealing a complex interplay of metabolic factors in the context of conditions such as diabetes and chronic wounds. In diabetes, elevated glucose levels trigger a cascade of metabolic alterations within epithelial cells, significantly impairing their regenerative capacity [111, 112]. Central to this impairment is mitochondrial dysfunction [113]. Dysfunctional mitochondria not only contribute to reduced energy production but also lead to increased oxidative stress, which can exacerbate cellular damage and hinder the normal processes required for effective wound healing and tissue regeneration.

Furthermore, growth factors, such as ciliary neurotrophic factor (CNTF), play a significant role in promoting epithelial stem/progenitor cell activation and wound healing [114]. By modulating cellular metabolism, these factors enhance key pathways that promote cell proliferation and migration. However, in diabetes, a deficiency of these growth factors is noted, thereby impeding the metabolic processes necessary for efficient epithelial regeneration [115]. This deficiency can disrupt the carefully balanced relationship between energy production and utilization, compromising the cell’s ability to undertake the energetically demanding process of tissue repair.

Moreover, calcium (Ca^2+^) homeostasis, a fundamental regulator of various metabolic pathways, emerges as a key factor influencing epithelial regeneration [113]. Imbalances in Ca^2+^ levels, often observed in metabolic disorders like diabetes, can perturb multiple cellular metabolic processes and signaling cascades [113]. By restoring disrupted metabolic signaling, researchers aim to improve the cell’s capacity to undergo essential metabolic transitions crucial for tissue repair.

In addition, keratinocytes play a significant role by migrating to cover the wound bed, proliferating, and ultimately restoring the skin barrier. This dynamic process requires significant energy, and keratinocytes rely on various metabolic pathways to meet these demands [16]. Central to their energy production is glycolysis, a process that generates ATP rapidly in the cytoplasm without the need for oxygen. While glycolysis is less efficient than mitochondrial OXPHOS in terms of ATP yield, it allows keratinocytes to meet their immediate energy needs, especially in the hypoxic environment of a wound [16].

In the early stages of reepithelialization, glycolysis becomes the predominant metabolic pathway due to the limited oxygen supply caused by impaired blood flow to the wound site. This metabolic switch, known as the “Warburg effect”, where cells favor glycolysis even in the presence of oxygen, is crucial for rapidly producing the energy and biosynthetic precursors needed for cell proliferation and migration [16, 116]. Glycolytic intermediates also serve as building blocks for nucleotide and amino acid synthesis, which are essential for DNA replication and protein synthesis during keratinocyte proliferation.

As the wound environment begins to stabilize and oxygen levels improve, keratinocytes gradually transition back to mitochondrial oxidative metabolism, particularly through the TCA cycle and OXPHOS, to support long-term tissue repair and differentiation [117]. Mitochondrial respiration becomes increasingly important for sustaining ATP levels during the later stages of reepithelialization, especially as keratinocytes differentiate and contribute to the formation of a new, functional epidermal layer.

In addition to ATP production, metabolic pathways such as the PPP play a key role in keratinocyte function during wound healing [118]. The PPP not only provides reducing equivalents in the form of NADPH, which helps combat oxidative stress, but also generates ribose-5-phosphate for nucleotide synthesis [118, 119]. This balance between energy production and redox homeostasis is critical, as the wound environment is often characterized by high levels of ROS, which can impede keratinocyte migration and function if not properly regulated.

## Metabolism-targeting therapies for diabetic wound

**Fig. 3:**
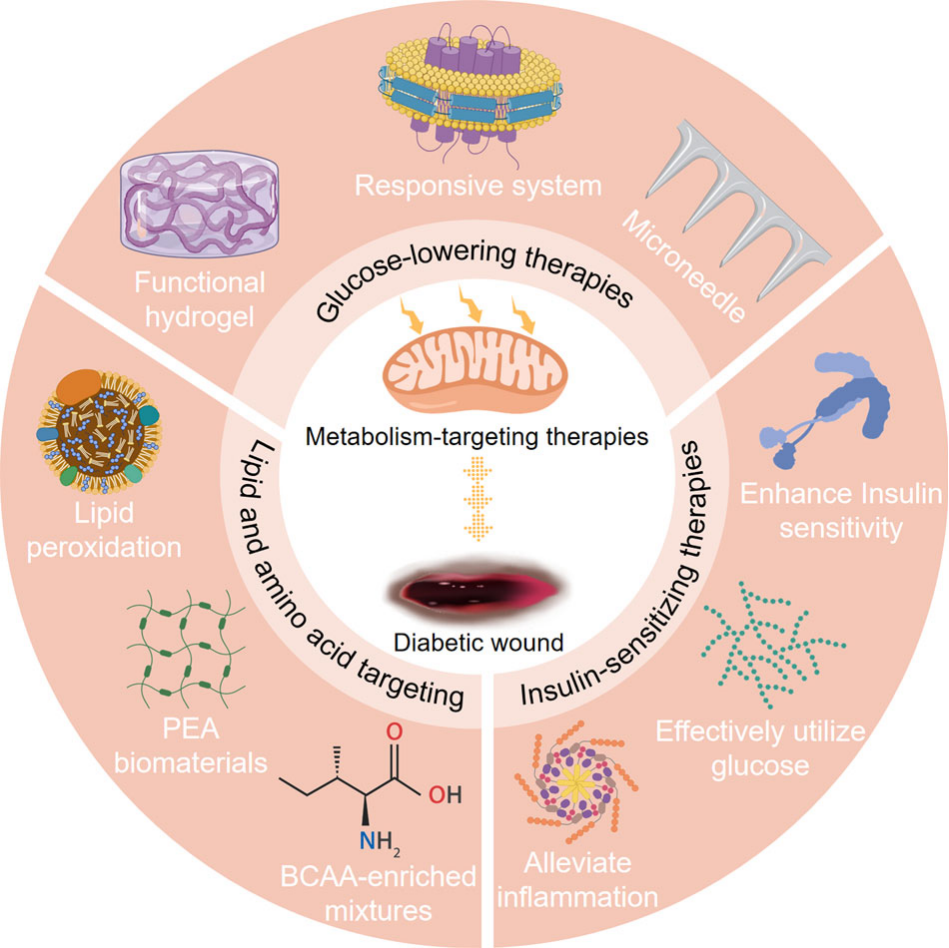
Illustration of the various therapeutic approaches aimed at addressing diabetic wounds through the modulation of metabolism. These approaches encompass strategies for reducing glucose levels, enhancing insulin sensitivity, and targeting lipids and amino acids. Created with MedPeer (https://www.medpeer.cn/).

Therapies targeting metabolism for diabetic wounds represent a promising approach, transitioning away from mere symptomatic treatment to addressing the underlying metabolic disturbances. Traditionally, wound care relied heavily on the optimal use of antibiotics often resulting in an oversight of the fundamental concern of diabetes-induced hyperglycemia. In contrast, metabolism-targeting therapies directly address the metabolic imbalances present in impaired wounds [60]. These advanced interventions include a wide range of approaches, such as glucose-lowering therapies, insulin-sensitizing therapies, and lipid and amino acid targeting therapies [120–122] (Fig. 3). By targeting the underlying metabolic dysregulations, these therapies are promising to not only facilitate wound healing but also to enhance overall clinical outcomes for individuals with diabetes.

Therapeutic approaches aimed at modulating metabolic processes in wound healing focus on restoring energy production, reducing oxidative stress, and enhancing cellular functions critical for tissue repair. In diabetic wounds, where metabolic dysfunction impairs healing, therapies that target mitochondrial function, such as resveratrol and metformin, aim to enhance ATP production by stimulating mitochondrial biogenesis and improving oxidative phosphorylation [123, 124]. These treatments promote essential cellular activities like keratinocyte migration and ECM production. Antioxidant therapies, including agents like *N*-acetylcysteine (NAC) and vitamin E, address the excess of ROS in diabetic wounds, reducing oxidative damage and preventing prolonged inflammation. Additionally, therapies that activate glycolysis, such as growth factor-based treatments like VEGF, provide rapid energy to cells in the hypoxic wound environment, facilitating early wound closure [60, 125].

However, several challenges persist. The complexity of wound healing requires precise timing and dosage to avoid disrupting essential cellular processes. For instance, while reducing oxidative stress is beneficial, excessive ROS scavenging could impair normal cell signaling [126]. Delivery of these metabolic modulators poses another challenge, as they should reach targeted cells like keratinocytes and fibroblasts without affecting surrounding tissue [127]. Nanotechnology-based delivery systems, such as nanoparticles and hydrogels, are being explored to improve the precision and efficacy of these treatments, though patient variability and the systemic nature of diabetic dysregulation complicate therapy outcomes [128]. Personalized approaches considering both the patient’s overall metabolic health and the specific characteristics of the wound may be necessary for optimal treatment.

### Glucose-lowering therapies

Glucose-lowering therapies for diabetic wounds have emerged as a transformative approach to address the serious challenges posed by chronic and non-healing wounds in individuals with diabetes [70]. The concept of glucose-responsive therapies represents a significant advancement in the treatment of diabetic wounds, emphasizing the potential for personalized and adaptive interventions. These therapies are designed to respond dynamically to the varying glucose levels within the wound microenvironment, offering an effective approach to enhance healing outcomes [129]. These therapies utilize innovative mechanisms and materials, including phenylboronic acid units and advanced drug delivery systems, to provide a dynamic framework for glucose regulation [129]. Importantly, these therapies seek to address the underlying cause of impaired wound healing in diabetes and its cascading effects on the cellular and molecular processes involved in tissue repair.

The molecular mechanisms underlying glucose-responsive therapies are complex yet promising. These therapies typically incorporate glucose-sensitive components that enable them to adapt to varying glucose concentrations within the wound. A critical element may include phenylboronic acid units, which experience reversible charge changes in response to fluctuations in glucose levels [130]. This innovative molecular design enables the therapeutic system to detect and respond appropriately to fluctuations in glucose levels within the wound microenvironment. When triggered by elevated glucose concentrations, these components can initiate a cascade of events, including glucose oxidation, hydrogen peroxide generation, or the release of insulin and glucagon [131]. These molecular mechanisms can effectively address various aspects of impaired wound healing associated with diabetes. For instance, the generation of ROS can help combat infection, while the release of insulin and glucagon can restore hormonal balance, which is essential for tissue repair [132]. These therapies thus offer a multifaceted approach to diabetic wound management. Consequently, reduced hyperglycemia enhances wound healing kinetics through decreased inflammation, promoted angiogenesis, and increased collagen production. [131, 133].

Treatment approaches for glucose-responsive therapies include a range of innovative modalities, such as hydrogel dressings, microneedle patches, and other advanced drug delivery systems [130, 131, 133]. These platforms are designed to utilize glucose-responsive components and molecular mechanisms, providing a dynamic, patient-centered approach to diabetic wound management. For example, hydrogel dressings can respond to fluctuations in glucose levels, releasing insulin, glucagon, antioxidants, or antimicrobial agents as needed to optimize the wound microenvironment [134]. Similarly, microneedle patches that incorporate glucose-responsive components are designed to functionally regulate the release of insulin and glucagon in a self-regulating manner, ensuring precise and timely hormonal regulation tailored to individual glucose dynamics [131]. These treatment modalities offer significant potential to transform the landscape of diabetic wound care, providing patients with effective, personalized, and adaptive solutions for managing these complex complications.

### Insulin-sensitizing therapies

Insulin-sensitizing therapies for diabetic wounds are a multifaceted approach aimed at addressing the challenges of impaired wound healing in individuals with diabetes [135]. Central to these therapies is the central role of insulin in regulating glucose metabolism and its vital contribution to tissue repair and regeneration. In diabetes, insulin resistance and metabolic dysfunction hinder the body’s ability to respond effectively to insulin, resulting in hyperglycemia and a myriad of clinical complications, including delayed wound healing. Insulin-sensitizing therapies seek to rectify this by restoring the body’s sensitivity to insulin, thus promoting wound healing [136].

Individuals with diabetes can anticipate several physiological improvements from these treatments. Primarily, they lead to a significant increase in insulin sensitivity, enabling cells throughout the body to utilize glucose more effectively. This enhancement helps mitigate the hyperglycemic state that can severely hinder wound healing [137]. In addition to improved glucose control, these therapies often lead to reduced inflammation at the wound site, addressing a common barrier to healing in individuals with diabetes [138]. By targeting the underlying cause of insulin resistance, these therapies effectively address the consequences of hyperglycemia, leading to tangible clinical benefits.

At the molecular level, insulin-sensitizing therapies utilize various mechanisms to achieve their aims. A key pathway involves the activation of peroxisome proliferator-activated receptor gamma (PPARγ), a nuclear receptor that is crucial for regulating insulin sensitivity [120]. Thiazolidinediones (TZDs), such as rosiglitazone, are medications that operate by activating PPARγ. Through this mechanism, they enhance insulin sensitivity, reduce inflammation, and ultimately promote wound healing. The molecular interaction between TZDs, PPARγ, and improved insulin sensitivity highlights their therapeutic potential in addressing diabetic wounds [137].

Another molecular factor implicated in these therapies is FGF1 [120]. FGF1 belongs to a family of growth factors involved in various physiological processes, including development, wound healing, and cardiovascular health. Interestingly, FGF1 has shown distinct linkage with the nuclear receptor PPARγ [120]. The interplay between FGF1 and PPARγ may be crucial for enhancing insulin sensitivity and promoting improved wound healing. Despite the limited clinical success of FGF1 and other growth factor-based treatments, these findings highlight the complex molecular network that drives insulin-sensitizing therapies.

From a clinical perspective, insulin-sensitizing therapies have significant consequences for individuals with diabetes. Perhaps the most tangible outcome is the acceleration of wound healing. Enhanced insulin sensitivity and better glycemic control create an environment conducive to faster and more effective wound repair. Further, the state of chronic inflammation in diabetic wounds is reduced, thereby contributing to improved healing outcomes. Moreover, these therapies extend their influence beyond wound healing, offering the potential for metabolic control in diabetes management.

Insulin-sensitizing therapies encompass a diverse array of treatment modalities for diabetic wounds. Medications such as TZDs, including rosiglitazone, are frequently used to improve overall insulin sensitivity [139]. Through the activation of PPARγ, the insulin resistance observed in diabetes is targeted, and a more conducive environment for wound healing is created. Focusing on the regional regulation of glucose metabolism rather than systemic adjustment, insulin release systems, including hydrogels and dressings, are designed to deliver insulin in a controlled and sustained manner directly to the wound site [140]. These systems support the regenerative processes essential for healing by maintaining optimal regional glucose levels at the wound site [141].

### Lipid and amino acid targeting therapies

Diabetic wounds often exhibit impaired healing due to underlying metabolic and inflammatory dysregulations. Among the lipid targeting therapies, Annexin A1 (ANXA1) has emerged as a key player [36]. ANXA1, known for its anti-inflammatory properties, contributes to inflammation resolution and improved metabolism. ANXA1 deficiency in diabetic mice has been shown to be associated with exacerbated renal injuries with albuminuria, mesangial matrix expansion, and tubulointerstitial lesions, alongside intrarenal lipid accumulation and mitochondrial alterations. The therapeutic potential of ANXA1 is further highlighted by the positive effects of Ac2-26, an ANXA1 mimetic peptide, which demonstrated efficacy against lipid toxicity in diabetic mice.

Of note, ferroptosis-targeting therapy has emerged as a promising approach to address the impaired wound healing observed in diabetes [142]. Diabetic wounds often exhibit persistent oxidative stress and inflammation, contributing to delayed healing. Ferroptosis, characterized by iron-dependent lipid peroxidation and cell death, has recently been recognized as a key player in this process. In diabetes, there is an accumulation of ROS and iron overload, creating an environment conducive to ferroptosis. Strategies aimed at inhibiting ferroptosis, such as via the upregulation of Nrf2, a transcription factor involved in antioxidant responses, have shown potential in delaying the progression of diabetic wounds [142]. Compounds like Ferrostatin-1, which specifically inhibit ferroptosis, have demonstrated their ability in ameliorating tissue damage and improving wound healing in diabetic animal models [142]. By targeting the ferroptotic pathway, these therapies aim to reduce oxidative stress, lipid peroxidation, and subsequent inflammation, thereby promoting more efficient wound healing in diabetic individuals.

Interestingly, adjunct topical administration of low-dose aspirin has been shown to significantly improve cutaneous wound healing in diabetic mice [143]. Chronic wounds in diabetic individuals are often characterized by sustained inflammation which is targeted by aspiring through the promotion of a phenotypic switch in wound macrophages towards an anti-inflammatory and pro-resolutive profile, marked by the release of the pro-resolving lipid mediator LXA4 [143]. This approach offers another promising strategy to mitigate chronic inflammation and facilitate wound healing in diabetic patients.

In amino acid targeting therapies, a family of biodegradable amino acid-based poly(ester amine) (PEA) biomaterials has been designed to inhibit the NOS pathway in macrophages, which has been associated with impaired wound healing in conditions like diabetes [144]. These biomaterials demonstrated both biocompatibility and the ability to modulate the immune response. In diabetic rat models, PEA biomaterials accelerated wound healing by creating a pro-healing wound microenvironment with improved reepithelialization, collagen deposition, and angiogenesis. Mixtures enriched with branched-chain amino acid (BCAA) have also shown promise in promoting wound healing by stimulating mitochondrial biogenesis in skin cells, thereby enhancing markers involved in tissue repair [145].

NAC, a mucolytic agent, has been explored for diabetic eye diseases, due to its combined mucolytic, antioxidant, and anti-inflammatory properties [146]. Previous studies have explored the potential of topical NAC treatments for a range of conditions, including corneal wounds, chemical injuries, keratitis, dry eye disease, and meibomian gland dysfunction [146, 147]. Additionally, cerium-containing *N*-acetyl-6-aminohexanoic acid compounds have demonstrated the potential to accelerate wound repair in diabetic animals by reducing oxidative stress, enhancing angiogenesis, and promoting tissue repair [148].

### Emerging therapies targeting metabolic pathways

One notable development is the use of topical insulin treatment [149]. Traditionally used to manage blood glucose levels, insulin has recently been recognized for its local effects on wound healing. Topical insulin stimulates the AKT signaling pathway, promoting cell proliferation, migration, and protein synthesis, all of which are vital for wound closure [150]. By enhancing glucose uptake and utilization in keratinocytes and fibroblasts, topical insulin improves cellular energy production and accelerates tissue regeneration in diabetic wounds.

Another emerging therapy involves the use of agents that target mitochondrial dysfunction, a hallmark of impaired wound healing in diabetes. Mitochondria-targeted antioxidants, such as mitoquinone (MitoQ), are being explored to reduce the excessive production of ROS that results from faulty oxidative phosphorylation [151]. These antioxidants help restore redox balance and protect cells from oxidative damage, which is critical in promoting proper cell function and reducing inflammation in the wound bed.

In addition to insulin and mitochondrial-targeted therapies, metabolic modulators like sodium-glucose cotransporter 2 (SGLT2) inhibitors, commonly used to treat diabetes, are being investigated for their potential to enhance wound healing [152]. By altering glucose metabolism and reducing hyperglycemia-induced oxidative stress, these inhibitors may help restore the metabolic flexibility of wound cells, particularly keratinocytes and immune cells.

Emerging nanotechnology-based delivery systems are also advancing metabolic modulation in wound healing [153, 154]. Nanoparticles and hydrogels can deliver drugs in a controlled manner, ensuring that metabolic modulators reach specific cells like keratinocytes or fibroblasts without affecting surrounding tissues. Together, these novel therapies offer promising solutions for addressing metabolic dysfunction and improving healing outcomes in diabetic wounds.

### Perspectives

Metabolic dysregulations play a crucial role in the onset and progression of diabetic wounds, a complex and common complication associated with diabetes. This shift towards therapies that target metabolic pathways represents a significant advancement in diabetic wound management. Despite significant advances in understanding diabetic wounds and their underlying metabolic mechanisms, several key knowledge gaps persist in the current literature. One major area of uncertainty is the incomplete understanding of how chronic hyperglycemia alters immune responses [155]. While it is recognized that hyperglycemia contributes to a pro-inflammatory environment, the specific molecular pathways and cellular interactions that lead to altered immune cell function remain inadequately defined [156]. For instance, the precise mechanisms by which elevated glucose levels affect the phenotypic and functional polarization of macrophages are not fully elucidated, complicating efforts to target inflammation therapeutically.

Furthermore, the long-term impact of dysregulated metabolism on various cell types involved in wound healing is an area that requires further exploration. While studies have highlighted the roles of endothelial cells and fibroblasts in tissue repair [157, 158], the effects of chronic metabolic stress on the behavior of other critical cell types, such as keratinocytes and pericytes, remain largely unexplored. Understanding these interactions is essential for developing comprehensive therapeutic strategies that can effectively restore normal wound-healing processes.

Moreover, current therapeutic approaches for managing diabetic wounds often have limitations that highlight the need for innovative solutions. Existing treatments, such as insulin therapy and wound dressings, may not adequately address the multifaceted nature of chronic wounds in diabetes. There is a lack of targeted therapies that specifically address the metabolic dysregulation and inflammation that characterize diabetic wounds. In addition, the development of novel biomaterials and nanotechnology-based strategies holds promise, yet the translation of these approaches into clinical practice is still in its infancy.

The global impact of diabetic wounds on both patients and healthcare systems is substantial [159, 160]. The chronic nature of diabetic wounds not only reduces the quality of life but also places a substantial economic burden on healthcare systems. As research in this area progresses, the future of diabetic wound care looks increasingly promising. We can anticipate a time when individuals with diabetes will have access to more effective, personalized, and adaptable treatment options. By targeting the underlying metabolic issues rather than merely addressing the symptoms associated with diabetic wounds, these therapies have the potential to reduce both the prevalence and severity of these wounds, providing hope for millions affected by this challenging condition.
